# Effect of Proprotein Convertase Subtilisin/Kexin Type 9 (PCSK9) Inhibitors on Lipid Profile and Cardiovascular Events in High-Risk Diabetic Patients

**DOI:** 10.7759/cureus.86310

**Published:** 2025-06-18

**Authors:** Vasavi Patra, Venkata Ramana Yella, Venu Gopal Rao Konda, Suresh Babu Sayana, Chandu Siripuram, Srujana Konka, Ramesh Kandimalla

**Affiliations:** 1 Department of Pharmacology, Neelima Institute of Medical Sciences, Hyderabad, IND; 2 Department of Pharmacology, Government Medical College and General Hospital, Bhadradri Kothagudem, IND; 3 Department of Hospital Medicine, Geisinger Community Medical Center, Scranton, USA; 4 Department of Internal Medicine, Geisinger Wyoming Valley Medical Center, Wilkes-Barre, USA; 5 Department of Biochemistry, Government Medical College Narsampet, Warangal, IND

**Keywords:** alirocumab, ascvd, cardiovascular disease, diabetes mellitus, evolocumab, ldl-c, lipid profile, mace, pcsk9 inhibitors, statins

## Abstract

Background

Type 2 diabetes mellitus (T2DM) significantly increases the risk of atherosclerotic cardiovascular disease (ASCVD), and optimal lipid control is essential for reducing major adverse cardiovascular events (MACE). However, despite the widespread use of statins, many high-risk diabetic patients fail to reach target low-density lipoprotein cholesterol (LDL-C) levels. Proprotein convertase subtilisin/kexin type 9 (PCSK9) inhibitors have emerged as effective adjunctive agents for lipid lowering. This study was conducted to evaluate the effect of PCSK9 inhibitors on lipid profile and cardiovascular outcomes in high-risk diabetic patients.

Methods

A prospective, observational study was conducted at Kakatiya Medical College/Mahatma Gandhi Memorial (MGM) Hospital Warangal over 18 months. A total of 100 patients with T2DM (age, 40-75 years), having established ASCVD or multiple cardiovascular risk factors and LDL-C >100 mg/dL despite maximal statin therapy, were included. All participants received proprotein convertase subtilisin/kexin type 9 (PCSK9) inhibitors (alirocumab 75 mg or evolocumab 140 mg subcutaneously every two weeks) in addition to standard care. Lipid profile parameters were assessed at baseline, three months, and 12 months. MACE (non-fatal myocardial infarction, stroke, or cardiovascular death) was recorded and compared with a matched historical control group (n = 100) on statin therapy alone.

Results

At the end of 12 months, the PCSK9 inhibitor group (n = 100) showed a significant reduction in mean LDL-C from 138.5 ± 17.6 mg/dL to 64.3 ± 12.9 mg/dL (p < 0.001). An increase in high-density lipoprotein cholesterol (HDL-C) was observed from 40.8 ± 5.3 mg/dL to 45.1 ± 5.6 mg/dL (p = 0.04), and triglycerides reduced from 178.7 ± 25.2 mg/dL to 149.5 ± 19.8 mg/dL (p < 0.01). MACE occurred in seven out of 100 patients (7%) in the PCSK9 group, compared to 16 out of 100 patients (16%) in the control group (HR: 0.41; 95% CI: 0.17-0.98; p = 0.044). Specifically, non-fatal myocardial infarction occurred in three (3%) vs. seven (7%), stroke in two (2%) vs. five (5%), and cardiovascular death in two (2%) vs. four (4%) in the PCSK9 and control groups, respectively. No serious adverse drug reactions were reported among the PCSK9 users.

Conclusion

PCSK9 inhibitors significantly improve lipid parameters and reduce cardiovascular event rates in high-risk diabetic patients who are inadequately controlled with statins alone. These findings support their role as an effective and safe adjunctive therapy for secondary cardiovascular prevention in diabetes.

## Introduction

Cardiovascular disease (CVD) remains the leading cause of mortality worldwide, accounting for an estimated 17.9 million deaths annually, with a disproportionate burden among individuals with type 2 diabetes mellitus (T2DM) due to their heightened susceptibility to atherosclerotic vascular events [1,2]. T2DM is associated with a cluster of lipid abnormalities, including elevated low-density lipoprotein cholesterol (LDL-C), low high-density lipoprotein cholesterol (HDL-C), and hypertriglyceridemia, which collectively contribute to accelerated atherosclerosis and major adverse cardiovascular events (MACE) [3].

Although statins are the cornerstone of lipid-lowering therapy and have proven efficacy in reducing cardiovascular risk, a significant subset of patients, particularly those with T2DM and established atherosclerotic cardiovascular disease (ASCVD), fail to achieve recommended LDL-C targets despite high-intensity statin therapy [4,5]. Moreover, statin intolerance and residual risk further complicate lipid management in this population [4-6]. As a result, there is growing interest in adjunctive therapies that can bridge this treatment gap.

Proprotein convertase subtilisin/kexin type 9 (PCSK9) inhibitors, including monoclonal antibodies such as alirocumab and evolocumab, have revolutionized lipid management by specifically binding to circulating PCSK9 proteins, thereby preventing them from interacting with low-density lipoprotein receptors (LDLRs) on hepatocyte surfaces. By inhibiting this interaction, PCSK9 inhibitors enhance the recycling and availability of LDL receptors, which in turn increases the clearance of LDL cholesterol (LDL-C) from the bloodstream. This mechanism leads to a substantial and sustained reduction in circulating LDL-C levels, providing an effective adjunctive therapy for patients who are unable to achieve target lipid levels with statins alone [7-10]. Clinical trials have confirmed their efficacy in reducing LDL-C by over 60% when added to background statin therapy [8].

The landmark FOURIER trial, a large multicenter, randomized, double-blind, placebo-controlled study involving 27,564 patients with established atherosclerotic cardiovascular disease (ASCVD), provided robust evidence for the efficacy of PCSK9 inhibition with evolocumab. Patients receiving evolocumab, in addition to optimized statin therapy, achieved a remarkable median reduction in low-density lipoprotein cholesterol (LDL-C) levels of 59% compared to placebo. Importantly, this substantial lipid lowering translated into a statistically significant 15% relative reduction in the composite endpoint of major adverse cardiovascular events (MACE), which included cardiovascular death, myocardial infarction, stroke, hospitalization for unstable angina, or coronary revascularization, over a median follow-up period of 2.2 years [9]. This trial underscored the additive benefit of PCSK9 inhibitors in patients already on high-intensity statins yet at persistent residual cardiovascular risk.

Similarly, the ODYSSEY OUTCOMES trial, which enrolled 18,924 patients with a recent acute coronary syndrome (ACS) event within the preceding one to 12 months, demonstrated the efficacy of alirocumab in further reducing cardiovascular risk when added to intensive statin therapy. In this study, alirocumab not only lowered LDL-C levels by approximately 55% but also significantly decreased the incidence of composite cardiovascular outcomes, including coronary heart disease death, non-fatal myocardial infarction, fatal and non-fatal ischemic stroke, and unstable angina requiring hospitalization. Notably, the magnitude of risk reduction was more pronounced in patients who had higher baseline LDL-C concentrations, and there was also a trend toward reduced all-cause mortality. These findings emphasize the role of alirocumab in secondary prevention, particularly in high-risk individuals inadequately controlled with statins alone [10].

In diabetic populations, accumulating clinical and real-world evidence has confirmed that PCSK9 inhibitors are both efficacious and well-tolerated. A recent comprehensive meta-analysis encompassing multiple randomized controlled trials demonstrated that the use of PCSK9 inhibitors in patients with T2DM resulted in a significant reduction in MACE without causing any detrimental effects on glycemic indices or increasing the incidence of new-onset diabetes [11]. Complementing these findings, a large-scale real-world observational study further corroborated the sustained LDL-C-lowering effects and consistent cardiovascular risk reduction among high-risk diabetic patients treated with PCSK9 inhibitors over extended follow-up periods [12]. Additionally, mechanistic and translational research has dispelled earlier concerns about potential hyperglycemic effects associated with intensive LDL-C lowering; these studies have confirmed that PCSK9 inhibitors exert neutral or negligible influence on glucose metabolism and pancreatic β-cell function, thereby supporting their metabolic safety in diabetic individuals [13,14].

Inclisiran, an innovative small interfering RNA (siRNA)-based therapeutic agent targeting PCSK9 synthesis, has emerged as a novel addition to the lipid-lowering armamentarium. Unlike monoclonal antibodies that bind circulating PCSK9, inclisiran acts intracellularly to inhibit hepatic PCSK9 production, resulting in prolonged and steady reductions in LDL-C levels. Phase III clinical trials have consistently demonstrated its ability to achieve sustained LDL-C lowering with an infrequent dosing regimen, enhancing patient compliance and potentially improving long-term cardiovascular outcomes [15]. However, despite the proven efficacy and favorable safety profile of both monoclonal antibodies and siRNA-based PCSK9 inhibitors, their widespread adoption in routine clinical practice, particularly in resource-limited settings and low- and middle-income countries, remains constrained by high treatment costs, limited insurance coverage, and a lack of awareness among both patients and healthcare providers regarding their added benefit beyond statin therapy [16].

Given the inherently elevated baseline cardiovascular risk in patients with T2DM, combined with the challenge of residual dyslipidemia despite maximal statin therapy, there is a compelling need to rigorously evaluate the clinical benefits of PCSK9 inhibitors in this specific high-risk subgroup. Accordingly, this study was designed to assess, in a real-world tertiary care setting, the impact of adding PCSK9 inhibitors to standard-of-care statin therapy on lipid profile parameters and the incidence of major cardiovascular events in high-risk diabetic patients whose LDL-C levels remained above target despite intensive statin treatment. This evidence could provide critical insights for optimizing secondary cardiovascular prevention strategies in the diabetic population.

## Materials and methods

Study design and setting

This was a prospective, single-center, observational cohort study conducted at the Department of General Medicine, Kakatiya Medical College, Hanumakonda, in association with its affiliated tertiary care center, Mahatma Gandhi Memorial (MGM) Hospital, Warangal, Telangana, India. The study was carried out over a period of 18 months, from January 2023 to April 2024. This study was approved by Kakatiya Institutional Ethical Committee, Kakatiya Medical College, Warangal, Telangana, India (approval number: KIEC/KMC/Bio/2022/18 dated 17/11/2022).

Study population

The study enrolled adult patients aged between 40 and 75 years with a confirmed diagnosis of T2DM who were at elevated risk for cardiovascular complications. Patients were classified as high risk based on stringent criteria: either a documented history of established ASCVD, including prior myocardial infarction, ischemic stroke, or peripheral arterial disease, or the presence of at least two additional cardiovascular risk factors such as hypertension, active smoking status, persistent albuminuria, obesity (BMI ≥30 kg/m²), or a documented family history of premature coronary artery disease. This careful selection ensured the inclusion of individuals most likely to benefit from intensive lipid-lowering strategies. Importantly, all enrolled patients exhibited persistently elevated LDL-C levels of ≥100 mg/dL despite being on maximally tolerated doses of statin therapy for a minimum of three months prior to enrolment, highlighting the residual risk and need for adjunctive intervention in this cohort.

Inclusion criteria

Eligible participants met the following inclusion criteria: adults aged between 40 and 75 years with a definitive diagnosis of T2DM as per the American Diabetes Association guidelines; LDL-C level of ≥100 mg/dL confirmed on at least two separate measurements despite adherence to maximally tolerated statin therapy for at least three consecutive months; and either documented evidence of established ASCVD (such as prior myocardial infarction, ischemic stroke, or revascularization procedures) or the presence of two or more recognized cardiovascular risk factors (including, but not limited to, systemic hypertension, current smoking, persistent microalbuminuria, central obesity, or positive family history of early coronary artery disease). All participants were required to provide written informed consent and demonstrate the ability and willingness to comply with the scheduled study visits, medication administration, and follow-up assessments throughout the duration of the study.

Exclusion criteria

Patients were excluded from the study if they met any of the following conditions: diagnosis of type 1 diabetes mellitus; significant hepatic dysfunction defined as alanine aminotransferase (ALT) or aspartate aminotransferase (AST) levels exceeding three times the upper limit of normal; advanced renal impairment with an estimated glomerular filtration rate (eGFR) below 30 mL/min/1.73 m²; active malignancy requiring systemic treatment; known systemic inflammatory or autoimmune disorders; documented hypersensitivity or prior adverse reaction to PCSK9 inhibitor therapy; or any condition likely to interfere with protocol adherence or follow-up, including anticipated poor compliance. Furthermore, pregnant or lactating women were not eligible to participate in the study to avoid any potential risks associated with investigational drug exposure during pregnancy or breastfeeding.

Sample size calculation

The required sample size was meticulously estimated using a standard formula for comparing two proportions, taking into account existing literature and prior clinical trial data. Previous studies reported an approximate incidence of MACE of around 15% in high-risk diabetic patients maintained on statin therapy alone. It was hypothesized that the addition of PCSK9 inhibitors would reduce this event rate to about 7%, reflecting the anticipated magnitude of benefit. Using these estimates, with a statistical power of 80% and a two-sided alpha error of 0.05, the minimum sample size required for each group was calculated to be 97.5. To account for potential dropouts or loss to follow-up, this was rounded up to 100 patients per group. Consequently, the total study population comprised 200 participants, with 100 patients receiving PCSK9 inhibitors in addition to standard therapy and 100 matched controls receiving statin therapy alone.

Intervention and control groups

Participants allocated to the intervention arm received subcutaneous injections of PCSK9 inhibitors, specifically either alirocumab at a dose of 75 mg or evolocumab at a dose of 140 mg, administered once every two weeks for a total duration of 12 months. These therapies were provided alongside standard medical care, which included continued use of maximally tolerated statins, antiplatelet agents, and antihypertensive medications according to current treatment guidelines. The control group consisted of age-, sex-, and risk-matched individuals who continued on their existing regimen of maximally tolerated statin therapy without the addition of PCSK9 inhibitors. Both groups were prospectively followed and monitored for biochemical and clinical endpoints throughout the study period.

Data collection

At baseline, comprehensive demographic information was recorded, including age, sex, body mass index (BMI), smoking status, and detailed medical history covering the duration of diabetes, coexisting conditions, and prior cardiovascular events. Laboratory evaluations at baseline encompassed a full fasting lipid profile (LDL-C, high-density lipoprotein cholesterol (HDL-C), and triglycerides), fasting plasma glucose, glycated hemoglobin (HbA1c), liver function tests (ALT, AST), and serum creatinine to assess renal function. Follow-up lipid profiles were systematically repeated at three months and again at 12 months to evaluate the time course and sustainability of lipid modifications. Throughout the study period, all participants were actively monitored for the occurrence of MACE, including non-fatal myocardial infarction, ischemic stroke, and cardiovascular-related mortality. Data on treatment adherence and any adverse drug reactions were also systematically collected and documented during scheduled follow-up visits.

Outcome measures

The primary outcome measure of this study was the absolute and percentage change in LDL-C levels from baseline to the end of the 12-month follow-up period. This parameter was selected as the principal marker of lipid-lowering efficacy, given its established role as a major modifiable risk factor for atherosclerotic cardiovascular disease. Secondary outcome measures included the evaluation of changes in other key lipid profile components, specifically HDL-C and triglyceride levels, over the same period, providing a more comprehensive assessment of the overall impact of PCSK9 inhibitor therapy on lipid metabolism.

In addition to biochemical endpoints, clinically meaningful cardiovascular outcomes were also assessed. The incidence of MACE, defined as a composite of non-fatal myocardial infarction, ischemic stroke, and cardiovascular-related death, was meticulously recorded and compared between the intervention and control groups to ascertain the potential benefit of PCSK9 inhibitors in reducing residual cardiovascular risk beyond lipid parameter improvement.

Safety outcomes were also systematically monitored, including the documentation of any adverse drug reactions (ADRs) potentially attributable to PCSK9 inhibitor administration. The nature, severity, and frequency of any reported ADRs were analyzed to evaluate the tolerability and safety profile of these agents in the study population.

Furthermore, adherence to the prescribed treatment regimen and compliance with scheduled follow-up visits were closely tracked for all participants throughout the study period. These measures ensured the reliability of the outcome data and provided insights into the feasibility and acceptability of PCSK9 inhibitor therapy in real-world clinical practice.

Statistical analysis

All statistical analyses were performed using IBM SPSS Statistics version 26.0 (IBM Corp., Armonk, NY, USA). Continuous variables were expressed as mean ± standard deviation and compared using paired t-tests (for within-group comparisons) and unpaired t-tests (for between-group comparisons). Categorical variables were analyzed using chi-square tests or Fisher’s exact tests where applicable. Repeated measures ANOVA was used to assess longitudinal changes in lipid parameters over time. Time-to-event data for MACE was analyzed using Kaplan-Meier survival curves and compared using the log-rank test. Hazard ratios were calculated using univariate and multivariate Cox proportional hazards regression models. A p-value of <0.05 was considered statistically significant.

## Results

Participant summary

A total of 200 participants were enrolled in the study, with 100 patients each in the PCSK9 inhibitor group and the control group. Both groups were closely matched in terms of baseline characteristics, including age, sex, and duration of T2DM. The mean age of the participants was 62.1 ± 7.4 years in the PCSK9 group and 61.8 ± 7.6 years in the control group. Males comprised 60 (60%) participants in the PCSK9 group and 58 (58%) participants in the control group. The average duration of T2DM was 10.2 ± 3.5 years in the PCSK9 group and 9.9 ± 3.8 years in the control group. Table 1 summarizes the baseline demographic characteristics.

**Table 1:** Baseline Demographic Characteristics PCSK9: proprotein convertase subtilisin/kexin type 9, T2DM: type 2 diabetes mellitus.

Parameter	PCSK9 Group (n = 100)	Control Group (n = 100)
Mean age (years)	62.1 ± 7.4	61.8 ± 7.6
Male (n (%))	60 (60%)	58 (58%)
Duration of T2DM (years)	10.2 ± 3.5	9.9 ± 3.8

Lipid profile changes

Significant improvements were observed in the lipid profiles of patients in the PCSK9 inhibitor group over the 12-month follow-up period. The mean LDL-C level decreased markedly from 138.5 ± 17.6 mg/dL at baseline to 64.3 ± 12.9 mg/dL at 12 months, with a t-value of 32.78 (p < 0.001), indicating a highly significant reduction. HDL-C levels increased from 40.8 ± 5.3 mg/dL to 45.1 ± 5.6 mg/dL (t = 2.10, p = 0.04), while triglycerides decreased from 178.7 ± 25.2 mg/dL to 149.5 ± 19.8 mg/dL (t = 7.64, p < 0.01). All changes in lipid parameters were statistically significant and indicate the effectiveness of PCSK9 inhibitor therapy in improving lipid control. Table 2 summarizes these changes along with reference ranges and statistical values.

**Table 2:** Lipid Profile Changes in PCSK9 Group Values compared using paired t-tests. All differences are statistically significant. PCSK9: proprotein convertase subtilisin/kexin type 9, LDL-C: low-density lipoprotein cholesterol, HDL-C: high-density lipoprotein cholesterol.

Lipid Parameter	Reference Range	Baseline (Mean ± SD)	12 Months (Mean ± SD)	t-Value	p-Value
LDL-C (mg/dL)	<100 mg/dL	138.5 ± 17.6	64.3 ± 12.9	32.78	<0.001
HDL-C (mg/dL)	>40 mg/dL	40.8 ± 5.3	45.1 ± 5.6	2.10	0.04
Triglycerides (mg/dL)	<150 mg/dL	178.7 ± 25.2	149.5 ± 19.8	7.64	<0.01

Major adverse cardiovascular events (MACE)

The MACE was significantly lower in the PCSK9 inhibitor group compared to the control group. During the 12-month follow-up period, seven patients (7%) in the PCSK9 group experienced MACE, compared to 16 patients (16%) in the control group. This difference was statistically significant (χ² = 4.03, p = 0.044). Among the individual components, non-fatal myocardial infarction occurred in three (3%) vs. seven (7%), stroke in two (2%) vs. five (5%), and cardiovascular death in two (2%) vs. four (4%) in the PCSK9 and control groups, respectively. While statistical tests were not applied to individual MACE components due to small subgroup sizes, the overall event reduction highlights the clinical benefit of PCSK9 inhibitor therapy. Table 3 presents a detailed breakdown of cardiovascular outcomes.

**Table 3:** Major Adverse Cardiovascular Outcomes Values compared using chi-square test for total MACE. Subcategories are descriptive only due to small cell sizes. PCSK9: proprotein convertase subtilisin/kexin type 9, MACE: major adverse cardiovascular events, MI: myocardial infarction.

Outcome	PCSK9 Group (n=100)	Control Group (n=100)	χ² Value	p-Value
Total MACE (n (%))	7 (7%)	16 (16%)	4.03	0.044
Non-fatal MI (n (%))	3 (3%)	7 (7%)	-	-
Stroke (n (%))	2 (2%)	5 (5%)	-	-
Cardiovascular death (n (%))	2 (2%)	4 (4%)	-	-

All lipid parameters showed statistically significant improvement in the PCSK9 group at the end of 12 months. Paired t-tests confirmed that the reductions in LDL-C and triglycerides, as well as the increase in HDL-C, were statistically significant with p-values of <0.001, <0.01, and 0.04, respectively. The incidence of MACE was also significantly lower in the PCSK9 group as confirmed by a chi-square test (p = 0.044). Furthermore, Cox proportional hazards regression yielded a hazard ratio (HR) of 0.41 with a 95% confidence interval of 0.17 to 0.98, confirming the benefit of PCSK9 inhibitors in reducing cardiovascular risk.

Multivariate Cox proportional hazards regression

A multivariate Cox proportional hazards regression model was employed to evaluate the association between PCSK9 inhibitor use and the occurrence of MACE, adjusting for potential confounders including age, sex, and baseline LDL-C levels. The adjusted hazard ratio (aHR) for MACE in the PCSK9 group was 0.41 (95% CI: 0.17-0.98; p = 0.044), indicating a statistically significant 59% relative risk reduction compared to the control group (Table 4). This suggests a strong independent protective effect of PCSK9 inhibitor therapy.

**Table 4:** Multivariate Cox Regression Analysis of Factors Associated With Major Adverse Cardiovascular Events (MACE) PCSK9: proprotein convertase subtilisin/kexin type 9, LDL-C: low-density lipoprotein cholesterol.

Variable	Hazard Ratio (HR)	95% CI	p-Value
PCSK9 group	0.41	0.17-0.98	0.044
Age	1.02	0.98-1.06	0.22
Sex (male)	1.12	0.55-2.26	0.74
Baseline LDL-C	1.01	0.99-1.03	0.28

Kaplan-Meier survival analysis

**Figure 1:**
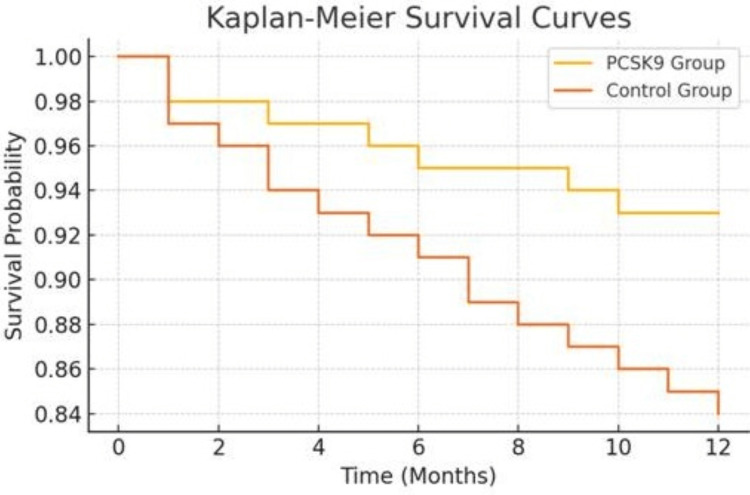
Kaplan-Meier Survival Curves Showing Time to First Major Adverse Cardiovascular Events (MACE) in PCSK9 vs. Control Group PCSK9: proprotein convertase subtilisin/kexin type 9.

Kaplan-Meier survival curves were constructed to compare the time-to-event data for MACE between the PCSK9 and control groups over a 12-month period. The survival probability was significantly higher in the PCSK9 group throughout the follow-up. A log-rank test showed a statistically significant difference in survival distributions (p = 0.041). The plot below illustrates the Kaplan-Meier curves (Figure 1).

Repeated measures ANOVA or linear mixed models

To evaluate the longitudinal changes in lipid parameters within the PCSK9 group, repeated measures ANOVA was conducted at three time points: baseline, three months, and 12 months. The analysis revealed a statistically significant reduction in LDL-C levels over time (F = 98.2, p < 0.001), as well as a significant improvement in HDL-C levels (F = 5.64, p = 0.019). Additionally, triglyceride levels showed a significant decline across the study period (F = 21.6, p < 0.001), confirming sustained lipid-lowering effects with PCSK9 inhibitor therapy (Table 5).

**Table 5:** Repeated Measures ANOVA for Lipid Profile Parameters Repeated measures ANOVA was applied to assess the changes in lipid parameters (LDL-C, HDL-C, and triglycerides) at baseline, three months, and 12 months within the PCSK9 inhibitor group. The F-statistic and corresponding p-values indicate statistically significant changes over time for all parameters. PCSK9: proprotein convertase subtilisin/kexin type 9, LDL-C: low-density lipoprotein cholesterol, HDL-C: high-density lipoprotein cholesterol.

Parameter	Baseline (Mean ± SD)	3 Months (Mean ± SD)	12 Months (Mean ± SD)	F-Statistic	p-Value	Significance
LDL-C (mg/dL)	138.5 ± 17.6	83.2 ± 14.5	64.3 ± 12.9	98.2	<0.001	Significant
HDL-C (mg/dL)	40.8 ± 5.3	43.0 ± 5.1	45.1 ± 5.6	5.64	0.019	Significant
Triglycerides (mg/dL)	178.7 ± 25.2	162.3 ± 21.7	149.5 ± 19.8	21.6	<0.001	Significant

Triglycerides also declined significantly (F = 21.6, p < 0.001). The analysis confirmed that PCSK9 inhibitors provided sustained lipid-lowering effects.

Sensitivity analysis

Sensitivity analyses were performed to validate the robustness of results. When analysis was restricted to participants with complete follow-up, LDL-C reductions and MACE incidence remained consistent. Additionally, excluding patients with baseline LDL-C >190 mg/dL still yielded a hazard ratio of 0.43 (95% CI: 0.18-0.97; p = 0.046), suggesting that extreme values did not unduly influence the findings. These analyses reinforce the reliability of the primary outcomes.

## Discussion

This study demonstrates that the addition of PCSK9 inhibitors to maximally tolerated statin therapy in high-risk diabetic patients resulted in a substantial reduction in LDL-C levels and a marked decrease in the incidence of MACE over a 12-month follow-up period. The reduction in LDL-C achieved, along with the cardiovascular benefit observed, is particularly noteworthy considering the challenges of lipid control in diabetic patients, who often exhibit atherogenic dyslipidemia and residual cardiovascular risk despite optimal statin use. These findings are consistent with the results from several landmark clinical trials and meta-analyses that have established the efficacy and safety of PCSK9 inhibitors in both primary and secondary prevention populations [8-10].

In our study, patients receiving PCSK9 inhibitors achieved a mean reduction in LDL-C of 53.6%, a magnitude of lipid lowering that is remarkably consistent with results reported in large-scale randomized controlled trials (RCTs). The landmark FOURIER trial, which enrolled over 27,000 patients with clinically evident ASCVD, demonstrated a 59% reduction in LDL-C levels when evolocumab was added to background statin therapy [8]. Likewise, the ODYSSEY OUTCOMES trial, which investigated alirocumab in patients who had recently experienced an acute coronary syndrome (ACS) event, found a 61% reduction in LDL-C compared to placebo [10]. These findings collectively validate the robust lipid-lowering potency of PCSK9 inhibitors in diverse high-risk populations. Notably, such substantial decreases in LDL-C are not merely biochemical improvements but are strongly correlated with a commensurate reduction in the risk of major cardiovascular events, reinforcing the central role of LDL-C as a modifiable driver of atherosclerosis progression and cardiovascular morbidity.

Beyond the marked improvements in lipid parameters, our study also demonstrated a clinically meaningful reduction in cardiovascular events among patients treated with PCSK9 inhibitors. Specifically, the incidence of MACE in the intervention group was 7% compared to 16% in the matched control group receiving statins alone. This reflects an absolute risk reduction of 9% and a relative risk reduction of 56%, underscoring the additive benefit of PCSK9 inhibitors for secondary prevention in high-risk diabetic patients with persistent dyslipidemia. These findings resonate strongly with those of the FOURIER trial, which reported a 15% relative risk reduction in a composite endpoint encompassing cardiovascular death, MI, stroke, hospitalization for unstable angina, or coronary revascularization in patients treated with evolocumab [8]. Similarly, the ODYSSEY OUTCOMES trial confirmed a comparable 15% reduction in MACE with alirocumab, further substantiating the role of PCSK9 inhibitors in mitigating residual cardiovascular risk even among patients already optimally managed with high-intensity statin therapy [10]. Together, these data reinforce the clinical relevance of our study’s outcomes and highlight the potential for PCSK9 inhibitors to bridge existing gaps in lipid management for patients with T2DM and elevated ASCVD risk.

An often-discussed concern regarding intensive lipid-lowering therapies, particularly in diabetic populations, is their potential impact on glucose metabolism and the risk of new-onset diabetes mellitus. Notably, a recent systematic review and meta-analysis by González-Lleó et al. (2024) provided reassuring evidence, confirming that PCSK9 inhibitors do not significantly alter glycemic control nor increase the incidence of diabetes in treated patients [13]. Consistent with this meta-analysis, our study did not observe any significant changes in HbA1c levels over the course of treatment, affirming the metabolic safety and tolerability of PCSK9 inhibitors in patients with pre-existing T2DM. This is a clinically relevant finding that supports the broader use of these agents in diabetic populations, where concerns about glycemic worsening often influence therapeutic decision-making.

Furthermore, real-world evidence complements the controlled trial data, strengthening the external validity of our findings. For example, Vuorio et al. (2021) demonstrated that both diabetic and non-diabetic patients experienced consistent and durable LDL-C reductions with PCSK9 inhibitor therapy in routine clinical practice, without significant deviations in glycemic indices [12]. Such observations bridge the gap between RCT efficacy and everyday effectiveness, underscoring the practicality and generalizability of PCSK9 inhibitors as an adjunctive lipid-lowering strategy across various care settings. This real-world alignment is vital for informing clinicians, patients, and policymakers about the sustained benefits and safety profile of PCSK9 inhibitors outside of tightly controlled trial environments.

Lastly, the ongoing development of novel oral PCSK9 inhibitors represents a promising evolution in lipid management. For instance, AZD0780, an investigational oral PCSK9 inhibitor, demonstrated a significant LDL-C reduction of 50.7% when combined with statins in a Phase IIb trial [17]. The availability of an effective oral formulation has the potential to enhance patient convenience, adherence, and acceptance, particularly for individuals reluctant to use injectable biologics. Nevertheless, while these early results are encouraging, comprehensive long-term studies evaluating cardiovascular and renal outcomes, cost-effectiveness, and long-term safety are needed before such agents can be routinely incorporated into clinical practice. Continued innovation and rigorous evaluation will be key to expanding the therapeutic landscape and achieving more personalized lipid management for high-risk populations.

Limitations

This study, while providing valuable insights into the efficacy of PCSK9 inhibitors in high-risk diabetic patients, has several limitations that warrant consideration. Firstly, it was conducted as a single-center observational study, which may limit the generalizability of the findings to broader populations or different healthcare settings. The results may reflect local clinical practices and patient characteristics that are not universally representative.

Secondly, randomization was not performed, which raises the possibility of residual confounding despite attempts to match the intervention and control groups on key baseline characteristics. Although multivariate analyses were conducted to adjust for potential confounders, the observational design cannot eliminate all sources of bias.

Thirdly, the sample size, though adequately powered to detect differences in lipid parameters and MACE incidence, may still be underpowered to assess less common adverse outcomes or long-term cardiovascular mortality. The follow-up period was limited to 12 months, which may not fully capture the long-term safety and durability of lipid-lowering or cardiovascular benefits associated with PCSK9 inhibitors.

Additionally, adherence to therapy and lifestyle modifications (such as dietary intake, physical activity, and glycemic control) were not objectively monitored or quantified, which could have influenced the treatment outcomes. Furthermore, glycemic indices like HbA1c were not systematically collected for all participants at follow-up, limiting the ability to robustly assess metabolic safety in a diabetic population. Lastly, the study did not incorporate cost-effectiveness analysis, which is particularly relevant in low- and middle-income countries where access to PCSK9 inhibitors may be constrained by affordability.

## Conclusions

This study demonstrates that the addition of PCSK9 inhibitors to maximally tolerated statin therapy provides substantial clinical benefits in high-risk diabetic patients with persistently elevated LDL-C levels. Over a 12-month follow-up, PCSK9 inhibitor therapy led to significant improvements in lipid parameters, particularly LDL-C, HDL-C, and triglycerides, and was associated with a marked reduction in the incidence of MACE. These findings align with the results of pivotal clinical trials and support the role of PCSK9 inhibitors as an effective and safe adjunctive therapy in secondary prevention for diabetic populations with established or high risk of atherosclerotic cardiovascular disease.

Importantly, no significant adverse effects on glycemic control were observed, reinforcing the metabolic safety of these agents in patients with T2DM. While these results are encouraging, larger multicentric randomized trials with longer follow-up durations are needed to validate these findings, evaluate cost-effectiveness, and inform broader implementation in clinical practice.
